# Intra-Session Repeatability of Static Pedobarometric Measurements: Analysis of Variability in Plantar Pressure Parameters and the Spatial Localization of the Center of Pressure and Peak Pressure

**DOI:** 10.3390/bioengineering13080940

**Published:** 2026-08-20

**Authors:** Lucia Bednarčíková, Teodor Tóth, Monika Michalíková, Patrícia Gajdošová

**Affiliations:** 1 Department of Biomedical Engineering and Measurement, Faculty of Mechanical Engineering, Technical University of Košice, Letná 1/9, 042 00 Košice, Slovakia; 2 Department of Languages, Technical University of Košice, Letná 1/9, 042 00 Košice, Slovakia

**Keywords:** pedobarometry, repeatability, center of pressure, peak pressure, plantar pressure parameters

## Abstract

Static pedobarometry is widely used to assess plantar pressure distribution and postural control; however, its clinical interpretation depends on measurement repeatability and reliability. The aim of our article is to evaluate the repeatability of plantar pressure parameters and the spatial localization of the center of pressure (CoP) and peak pressure (PP) during static bipedal standing. Five healthy adults underwent 30 repeated static measurements using the Sidas Press-Cam platform under standardized laboratory conditions. Analyzed parameters included contact area, mean pressure, peak pressure, and the center of pressure coordinates. Repeatability was assessed using intraclass correlation coefficients (ICC), standard error of measurement (SEM), and Bland–Altman analysis. Contact area showed high single-measure repeatability (ICC (2, 1) = 0.90). Peak pressure and mean pressure demonstrated moderate repeatability for single trials but excellent repeatability when averaged (ICC (2, k) = 0.97–0.996). The center of pressure exhibited greater mediolateral than anteroposterior variability. Peak pressure localization remained stable within 3 SD and predominantly on the dominant limb. Averaging multiple trials enhances measurement repeatability, suggesting that repeated static pedobarometric assessments may provide more consistent estimates of plantar pressure parameters. However, these findings should be interpreted as preliminary evidence from an intra-session repeatability study due to the limited sample size.

## 1. Introduction

Plantar pressure analysis using pedobarometric systems is an established method for objective assessment of foot load distribution during standing [1,2]. Static bipedal eyes-open measurements enable quantification of parameters such as peak pressure, mean pressure, contact area, and center of pressure, reflecting instantaneous body weight distribution between lower limbs. These parameters are widely used in orthopedic diagnostics and rehabilitation monitoring. However, their interpretation depends on understanding measurement variability and reliability [2]. Plantar pressure distribution arises from biomechanical, neuromuscular, and morphological factors and may vary slightly between trials [3,4]. Therefore, assessing intra-subject repeatability is essential for clinical interpretation, allowing separation of true postural variability from measurement-related effects.

Previous studies have demonstrated that static plantar pressure parameters can achieve moderate to high repeatability across different pedobarometric platforms. Alfaro-Santafé et al. evaluated repeated measurements during natural standing and reported good to very good repeatability of static parameters, while emphasizing that averaging multiple measurements improves the reliability of individual plantar load estimates [1]. Similarly, Izquierdo-Renau et al. demonstrated moderate to excellent intra- and inter-session reliability, with coefficients of variation not exceeding 12% [2]. Cobos-Moreno et al. reported high reliability for peak pressure, mean pressure, and contact area measurements using the Podoprint platform [5]. Collectively, these findings support the reproducibility of conventional plantar pressure parameters under controlled measurement conditions, while also demonstrating that measurement reliability is influenced by the repeated-measurement protocol. 

Lalande et al. [6] further examined plantar pressure distribution during quiet bipedal standing in a large sample of healthy individuals and established normative values for peak pressure, mean pressure, and contact area. Their findings demonstrated that these parameters can provide stable and reproducible outcomes under standardized conditions and highlighted the importance of consistent measurement protocols. However, the reproducibility of pressure magnitude does not by itself characterize whether the spatial organization of plantar loading remains stable across repeated trials. Parameters describing where the load is concentrated, particularly the position of the center of pressure and the localization of peak pressure, may provide complementary information on trial-to-trial variability that is not captured by pressure magnitude alone.

The need to consider variability beyond individual pressure values is also supported by broader methodological and clinical evidence. Mesci [7] emphasized that plantar pressure variables are influenced by both physiological and technical factors and that repeatability and reliability are prerequisites for clinically meaningful interpretation of pedobarometric measurements. Mirando et al. [8], using repeated measurements during quiet stance, observed inter-trial variability in regional peak pressure and overall pressure distribution, further indicating that repeated trials are necessary to distinguish stable loading characteristics from natural trial-to-trial variation. Together, these findings suggest that reliability assessment should address not only the magnitude of plantar pressure parameters but also the spatial behavior of plantar loading during repeated quiet standing.

In addition to methodological aspects, previous studies have shown that demographic and anthropometric characteristics, including sex and age, may influence plantar pressure distribution and should therefore be considered when interpreting pedobarometric measurements [9,10].

Despite the available evidence supporting the repeatability of static pedobarometric measurements, an important methodological question remains regarding how comprehensively repeatability should be characterized. Conventional reliability studies predominantly quantify the consistency of pressure-related parameters, such as peak pressure, mean pressure, and contact area. However, similar values across repeated trials do not necessarily imply identical spatial organization of plantar loading. The center of pressure and the location of peak pressure represent complementary spatial characteristics that may vary even when pressure magnitudes remain relatively stable. A combined assessment of conventional plantar pressure parameters and the spatial variability of center of pressure (CoP) and peak pressure (PP) within the same repeated-measurement protocol may therefore provide a more comprehensive description of within-subject repeatability during quiet standing.

Furthermore, repeatability based on a small number of trials primarily provides an estimate of agreement between individual measurements, whereas a larger series of repeated observations allows the distribution and spatial dispersion of within-subject variability to be characterized in greater detail. In the present pilot study, 30 repeated measurements per participant were therefore used to examine not only conventional reliability indices but also the trial-to-trial spatial dispersion of CoP and PP positions. This design enables pressure magnitude and spatial localization to be evaluated as complementary dimensions of repeatability under the same standardized intra-session conditions.

Therefore, the aim of this pilot intra-session study was to characterize within-subject repeatability of static pedobarometric assessment across 30 repeated measurements by integrating conventional plantar pressure parameters with spatial measures of plantar loading. Specifically, repeatability was evaluated for peak pressure, mean pressure, and contact area, together with the trial-to-trial spatial variability of CoP coordinates and PP localization during quiet bipedal standing with eyes open. The methodological contribution of this study lies in evaluating the magnitude and spatial organization of plantar loading as complementary dimensions of repeatability within a single repeated-measurement protocol. We hypothesized that conventional plantar pressure parameters would show consistent values across repeated measurements and increased reliability when multiple trials were averaged, whereas CoP and PP positions would exhibit limited spatial dispersion reflecting natural within-subject postural variability.

## 2. Materials and Methods

### 2.1. Participants

Five subjects (2 males, 3 females) aged 24 to 40 years participated in the study, with a mean age of 31 ± 6.56 years, height of 171 ± 8.98 cm, weight of 69.86 ± 14.49 kg and body mass index (BMI) of 22.57 ± 3.08 kg/m^2^ (range: 17.00–25.01 kg/m^2^). According to the World Health Organization classification, three participants had normal body weight, one was underweight, and one was slightly overweight. Foot size ranged from EU 38 to EU 44, and all participants were right-leg dominant, with limb dominance determined as the preferred leg used to kick a ball. All participants had physiologically normal foot arches without mild structural or functional foot deformities, such as hallux valgus, lesser toe deformities (e.g., hammer or claw toes), or noticeable leg length asymmetry, ensuring a homogenous sample of healthy feet. The alignment of the ankle joint, heel, and knee joint was in a neutral physiological position in all subjects and there was no history of lower-limb musculoskeletal injuries. The subjects participate in sports on a regular recreational physical activity level. 

Due to the exploratory nature of this investigation, the study was designed as a pilot intra-session repeatability assessment focusing on within-subject measurement variability rather than population-level reliability estimation. None of the participants had orthopedic or neurological disorders affecting the lower limbs, and all provided written informed consent prior to the commencement of the measurements. The study was conducted in accordance with the principles of the Declaration of Helsinki. 

### 2.2. Instrumentation

Measurements were performed using a static test on the Sidas Press-Cam pedobarometric platform, version 5.0 (SIDAS, Voiron, France). The active sensing area is 400×400 mm, with a sensor size (length/width) of 10 × 10 mm, a minimum/maximum pressure range per sensor from 0.4 to 100 N, and an acquisition frequency up to 100 images/s. The platform operates with Presscam V7.8 SD software (SIDAS, France). All measurements were conducted under laboratory conditions at a stable temperature on a level floor. Before data collection, participants were familiarized with the measurement procedure to ensure consistent execution of the testing protocol.

Thirty repeated measurements were performed for each participant. Each measurement lasted 10 s. Between consecutive trials, participants stepped off the platform and rested for 3 min before repositioning themselves for the subsequent measurement. This procedure was implemented to minimize the potential effects of fatigue, postural adaptation, and prolonged standing on measurement repeatability.

During each trial, participants stood barefoot on the pedobarographic platform in a natural, self-selected upright posture. Foot position, including foot angle and stance width (inter-foot distance), was not standardized in order to reflect each participant’s habitual standing posture. However, participants were instructed to adopt the same comfortable stance during each repeated measurement to minimize within-subject variability associated with changes in foot placement. The upper limbs were maintained in a neutral position alongside the trunk, and participants were instructed not to consciously modify their posture or redistribute their body weight. Throughout the measurement, participants were instructed to look straight ahead at a fixed point positioned at eye level in front of them to minimize unnecessary head and body movements. Measurements were initiated only after participants had assumed a stable standing position.

The outputs of the static test included color-coded pressure maps of plantar loading and quantitative data regarding total contact area (AC) of both feet, as well as individual limb contact areas (ACr, ACl). Additionally, peak pressure (PP) and its localization, the mean pressure (MP) of both feet, and the total center of pressure (CoPt) along with individual limb centers of pressure (CoPr, CoPl) were recorded.

### 2.3. Analysis of CoP and PP Parameters

Information regarding the center of pressure (CoP) position is not provided by the device in the form of numerical coordinates; instead, it is available exclusively as a visual representation within the color-coded pressure map for both feet simultaneously, as well as for each foot individually. Consequently, it was necessary to establish a reference Cartesian coordinate system to enable the quantitative determination of the CoP and peak pressure (PP) positions. The active sensing area dimensions (40 × 40 cm), as specified by the manufacturer, were used as the scale.

A base of support was defined on the color-coded plantar load map, serving as the reference frame for the coordinate system. The lower boundary of the base of support was designated as the x-axis, with the origin (0, 0) defined at the intersection of the lower boundary and the left lateral boundary of the base of support. The y-axis passed through this point, oriented perpendicularly to the x-axis.

Coordinate determination was consistently performed by the same investigator following a standardized procedure. Accordingly, the coordinates of individual centers of pressure were manually obtained for all 30 measurements of a single subject. Normalization or transformation of the base of support (e.g., scaling all bases to a uniform size or centering the coordinate system) was not required in this case, as all measurements pertained to a single subject. Therefore, deviations in the center of pressure position reflect actual differences in weight distribution and subtle changes in posture, rather than variations between different sizes of the base of support.

The position of peak pressure (PP) was analyzed within the area of both feet across 30 repeated measurements. As peak pressure in an individual measurement is always localized on either the left or the right foot, the frequency of its occurrence on each foot was evaluated across all repeated trials. Further spatial analysis included only those PP positions that occurred on the same foot in ≥60% of the repeated measurements, indicating a predominant side of peak pressure occurrence. The 60% threshold was selected as a methodological criterion to identify a stable pattern of peak pressure localization across repeated measurements while reducing the influence of random fluctuations in load distribution. Subsequently, the mean position of peak pressure and the standard deviation (SD) were calculated to characterize its typical spatial localization and variability.

### 2.4. Statistical Analysis

Statistical analysis was performed independently for peak pressure, mean pressure, and contact area, as well as for the base of support size and the positions of the CoP and PP. Data processing was conducted using Microsoft Excel for Microsoft 365 MSO (64-bit version 2607 build 16.0.20228.20190) (Microsoft Corporation, Redmond, WA, USA) and R 4.6.1 statistical software (R Foundation for Statistical Computing, Vienna, Austria), with the level of statistical significance set at *p* < 0.05.

The dataset consisted of 150 observations obtained from 30 repeated measurements in each of five participants. Because repeated measurements were nested within participants, all repeatability analyses were performed by considering repeated trials as repeated observations of the same subject.

Descriptive statistics included the mean, standard deviation (SD), median, and coefficient of variation (CV), calculated as

(1) $$CV=\frac{SD}{Mean}\times 100\%$$

For visualization of the spatial dispersion of CoP and PP, repeated coordinates were assumed to follow an approximately normal distribution. One- and two-standard-deviation regions (k = 1 and k = 2) were used to illustrate the expected spatial variability, with k = 2 encompassing approximately 95% of observations under the assumption of normality.

Measurement repeatability was evaluated using the intraclass correlation coefficient (ICC) based on a two-way random-effects model with absolute agreement. ICC (2, 1) was used to assess the repeatability of a single measurement, whereas ICC (2, k) was used to estimate the repeatability of the average of 30 repeated measurements. Variance components were obtained from a two-way random-effects analysis of variance (ANOVA), with participants treated as random effects and repeated measurements considered repeated observations within each participant. The model assumes normally distributed residuals, homogeneity of variance, and independent measurement errors. Ninety-five percent confidence intervals (95% CI) were calculated for all ICC estimates.

The standard error of measurement (SEM) was calculated as:

(2) $$SEM=SD\times \sqrt{\left(1-ICC\left(2,1\right)\right)}$$

where SD represents the standard deviation of the measurements and ICC (2, 1) represents the single-measurement repeatability coefficient.

Agreement between repeated measurements was evaluated using Bland–Altman analysis. All pairwise combinations of repeated measurements within each participant were generated, and for each pair the mean of the two measurements was plotted against their difference. The mean difference between paired measurements was considered the systematic bias. The 95% limits of agreement (LoA) were calculated as

(3) $$LoA=bias\pm 1.96\times {SD}_{difference}$$

where bias represents the mean difference between paired measurements and SD_difference_ is the standard deviation of the paired differences.

Bland–Altman plots were visually inspected for the presence of systematic trends or proportional bias across the measurement range. Because multiple pairwise comparisons originated from repeated measurements within the same participant, the Bland–Altman analysis was interpreted as a descriptive assessment of agreement rather than an inferential statistical test.

The spatial repeatability of CoP and PP localization was evaluated by calculating the mean coordinates and standard deviations of repeated measurements for each participant. Diagrams were constructed to visualize the dispersion of measured positions, with one- and two-standard-deviation regions representing the expected spatial variability of repeated measurements.

## 3. Results

The average size of the base of support ranged from 500.51 to 693.75 cm^2^ (SD = 15.99–47.34 cm^2^). The highest variability in the base of support configuration between repeated measurements was observed in Subject 1 (SD = 47.34 cm^2^) and Subject 4 (SD = 37.05 cm^2^). A moderate level of variability during postural reconfiguration was exhibited by Subject 2 (SD = 23.46 cm^2^), while Subjects 5 (SD = 15.99 cm^2^) and 3 (SD = 19.85 cm^2^) showed the most stable foot positioning.

In relation to the base of support, the actual contact area (AC) was analyzed, ranging from 186.13 ± 8.40 cm^2^ (Subject 5) to 266.13 ± 10.81 cm^2^ (Subject 4). Detailed descriptive statistics of the plantar parameters are presented in Table 1.

The mean value of peak pressure (PP) for all subjects was 825.9 ± 85.3 g/cm^2^, with individual coefficients of variation (CV) ranging from 5.8% to 8.8%. 

The mean value of mean pressure (MP) across all subjects was 328.3 ± 22.6 g/cm^2^, with individual CV values ranging from 3.3% to 6.7%.

Table 2 presents the mean coordinate values for the total center of pressure (CoPt), as well as for the right foot (CoPr) and left foot (CoPl), along with the corresponding standard deviations for each subject. The analysis revealed a systematically higher variability (SD) in the medio-lateral direction (x-axis) compared to the antero-posterior direction (y-axis) for all subjects.

**Figure 1:**
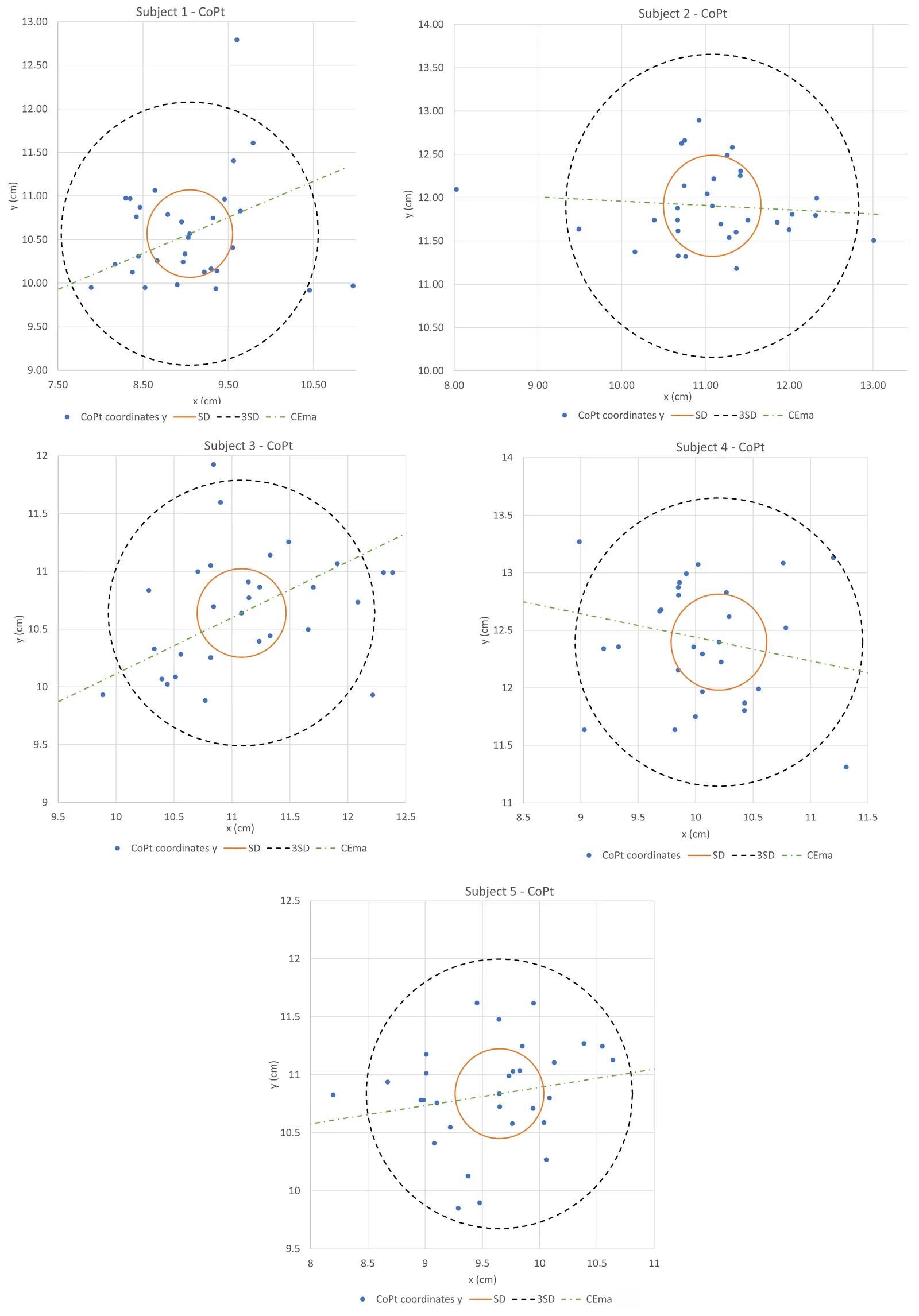
Spatial variability of total center of pressure (CoPt) coordinates during 30 repeated static measurements for each participant. Blue points represent individual CoPt positions, the inner circle indicates the area within one standard deviation (1SD), the outer circle represents the area within three standard deviations (3SD), and the dashed line indicates the confidence ellipse of the mean CoP position (CEma). The spatial distribution of CoP coordinates illustrates the variability in CoP location in the antero-posterior (AP) and medio-lateral (ML) directions across repeated measurements.

Three graphs illustrating the spatial variability of center of pressure (CoP) positions were generated for each participant (n = 5): for both feet (CoPt) (Figure 1), right foot (CoPr), and left foot (CoPl). The analysis included 30 repeated static measurements per participant. All recorded CoP positions were located within the three-standard-deviation (3SD) range, with the majority of measurements concentrated within the one-standard-deviation (1SD) range. These results demonstrate the consistency and spatial variability of CoP location across repeated static measurements in the antero-posterior (AP) and medio-lateral (ML) directions.

**Figure 2:**
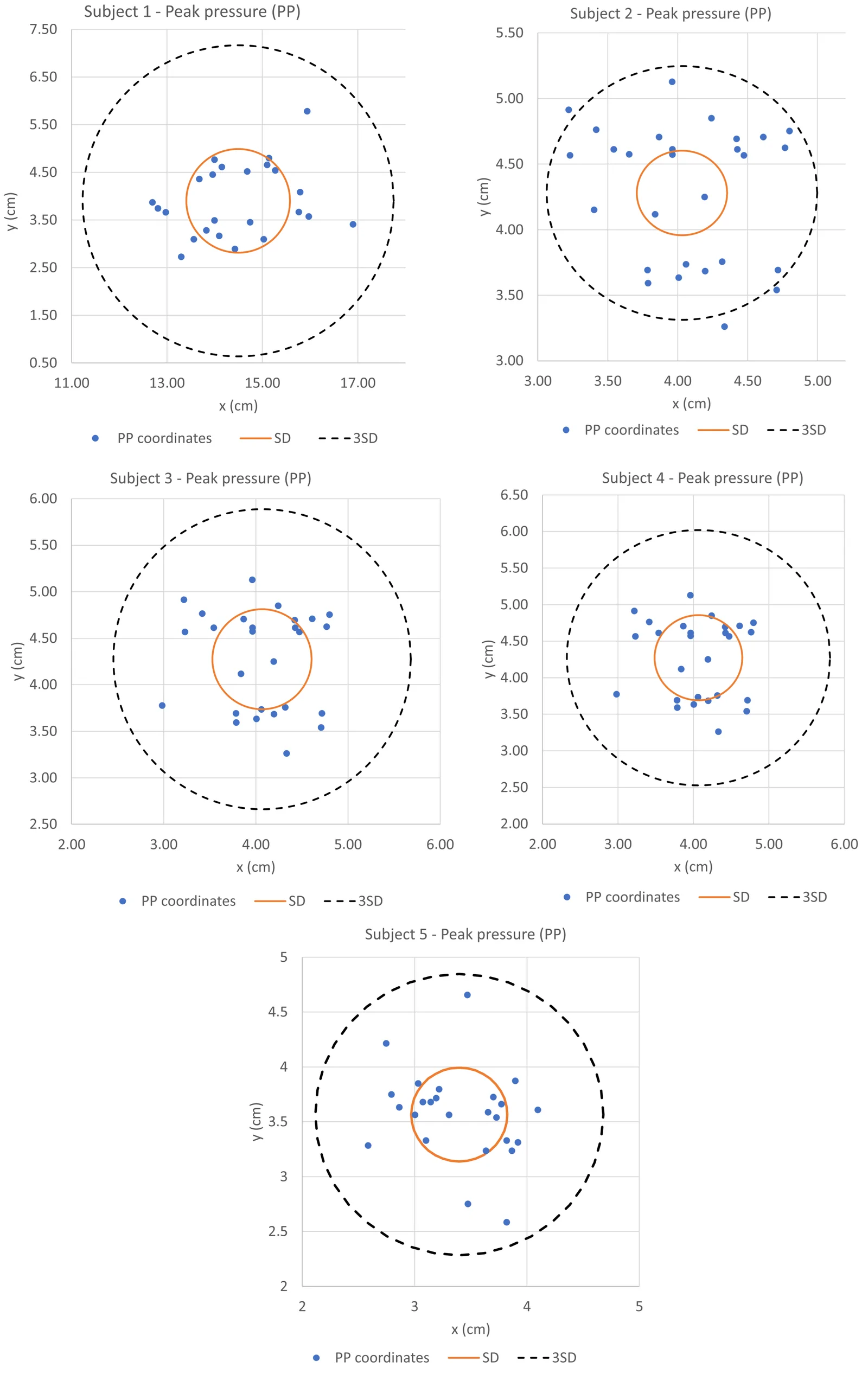
Spatial variability of peak pressure (PP) point localization on the predominant side of peak pressure occurrence during 30 repeated static measurements. Blue points represent individual PP coordinates, the orange circle indicates the area within one standard deviation (1SD), and the dashed circle represents the area within three standard deviations (3SD). The distribution of PP coordinates illustrates the repeatability and natural spatial variability of peak pressure localization across repeated measurements.

The analysis of the peak pressure (PP) position demonstrated the presence of a predominant loading pattern. The side showing a higher frequency of PP occurrence accounted for 63% to 97% of all measurements. The majority of PP points were located within the one-standard-deviation (SD) area, indicating a consistent spatial localization of PP during repeated measurements (Figure 2).

### Repeatability and Absolute Agreement of Measurements

The repeatability parameters and error estimates for the observed variables are summarized in Table 3. Measurement repeatability was evaluated using the intraclass correlation coefficient (ICC) to quantify the consistency of repeated measurements within subjects. The repeatability of a single measurement was moderate to high (ICC (2, 1) = 0.53–0.90, depending on the specific parameter), while the repeatability of the mean of 30 measurements reached very high values (ICC (2, k) = 0.97–0.996).

The standard error of measurement (SEM) was approximately 58.69 g/cm^2^ for peak pressure, 13.82 g/cm^2^ for mean pressure, and 5.04–9.58 cm^2^ for the contact area, indicating a low absolute measurement error.

**Figure 3:**
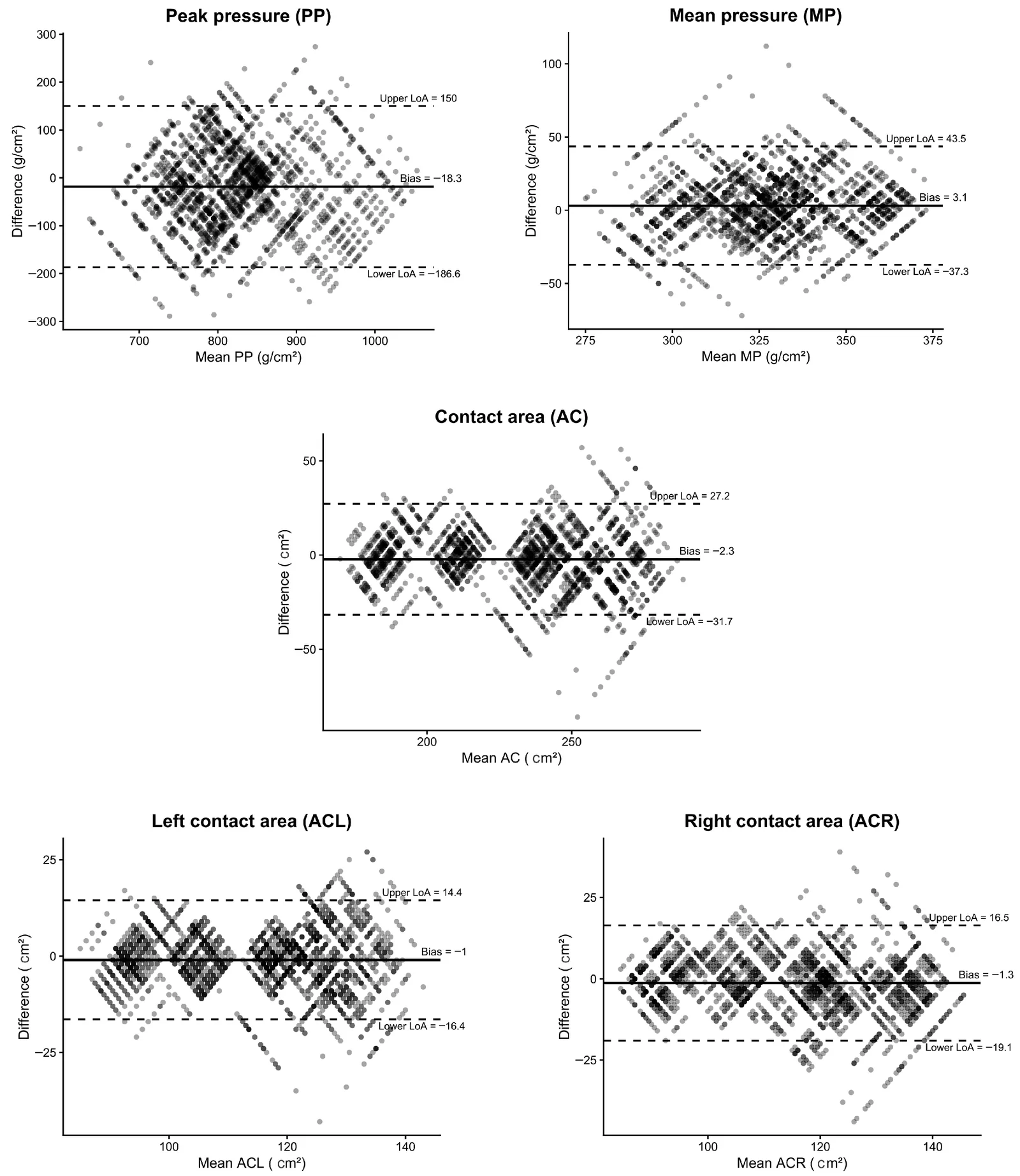
Bland–Altman plots showing agreement between repeated measurements of peak pressure (PP), mean pressure (MP), actual contact area (AC), actual left contact area (ACL), and actual right contact area (ACR). The solid horizontal line represents the mean difference (bias), while the dashed lines indicate the 95% limits of agreement (LoA = bias ± 1.96 × SD of the paired differences). Most paired differences were distributed within the limits of agreement, with no apparent proportional bias across the measurement range. Peak pressure exhibited the widest limits of agreement, reflecting greater variability compared with the remaining plantar pressure parameters.

Bland–Altman analysis demonstrated a low systematic bias between repeated measurements for all evaluated parameters. The majority of differences were distributed within the 95% limits of agreement, indicating stable measurement repeatability. No evident proportional bias or systematic trend related to the magnitude of measured values was observed (Figure 3).

## 4. Discussion

The aim of this pilot intra-session study was to assess the intra-session repeatability of plantar pressure parameters, including center of pressure (CoP) localization and peak pressure (PP) position during static standing. The results indicate that although human posture is a dynamic process characterized by inherent variability, averaging repeated measurements reduces intra-session variability and improves the consistency of the obtained plantar pressure estimates [1,2,3,4,5,6,7,11,12,13,14,15].

We found that contact area (CA) is the most stable parameter (ICC (2, 1) > 0.90), which is consistent with technical analyses of pedobarometric measurements [1]. The relatively low variability in contact area may reflect its dependence on foot morphology and its lower sensitivity to repeated postural adjustments. These findings are consistent with the results reported by Izquierdo-Renau et al. [2]. The stability of the contact area likely reflects its dependence on the morphological characteristics of the foot, which exhibit minimal variation between measurements.

Conversely, peak pressure (PP) exhibited substantial variability across individual measurements (ICC (2, 1) = 0.53) [6,7,8,13,16]. The repeatability increased significantly (ICC (2, k) = 0.97) using the average of 30 measurements, which aligns with the latest recommendations emphasizing the need for the aggregation of multiple trials [1,6,7,8,11,12,14,16]. In terms of clinical application, a single measurement may be prone to error [13]; however, a minimum of three trials should provide an ICC (2, 1) of approximately 0.75, which is considered to represent good repeatability [6,7,15]. Similarly, Alfaro-Santafé et al. [1] demonstrated that the mean value of multiple measurements significantly enhances the repeatability of static pressure parameters, thereby supporting the methodological approach adopted in this study. The relatively lower repeatability of a single peak pressure measurements may be attributed to its localized character and sensitivity to subtle variations in postural stability and muscle activation [13,16]. This phenomenon has been repeatedly documented in methodological studies, which highlight the increased variability of regional pressure parameters compared to global loading indices [16]. The pressure values obtained in this study are consistent with reference databases for the healthy population. These findings suggest that averaging multiple trials may improve the consistency of plantar loading estimates in healthy subjects by reducing the influence of individual measurement fluctuations [6,7,8,12,15,16].

The mean pressure (MP) parameter exhibited moderate relative reliability for a single measurement (ICC (2, 1) = 0.63), while the mean of multiple measurements reached an ICC (2, k) = 0.98, indicating excellent reproducibility. In this dataset, averaging multiple measurements contributed to achieving higher ICC values, suggesting improved consistency compared with single trials. The lower variability of mean pressure compared to peak pressure is likely related to its integrative character, as it represents the mean load across a larger plantar contact area and is less sensitive to local fluctuations [16].

Analysis of the spatial distribution of the center of pressure (CoP) (Figure 2) indicated a tight clustering of values around the mean position [7]. Similar repeatable patterns in CoP positioning during repeated measurements were reported by Alfaro-Santafé et al. [1], who highlighted the good reproducibility of global postural parameters during static standing. An interesting finding is the systematically higher variability along the x-axis (medio-lateral direction) compared to the y-axis [6,7]. The angle between the x-axis and the major axis of the confidence ellipse (CEma) ranges from −11.60° to 25.90° (Figure 2). Balance maintenance in the antero-posterior direction is more effectively controlled by the calf muscles (m. triceps surae), whereas the body balances in the medio-lateral direction using the foot invertors and evertors, leading to a wider dispersion of values (i.e., medio-lateral sway) [1,7].

An additional observation of this study was the tendency for peak pressure (PP) to occur repeatedly on the same limb across measurements (Figure 2). While PP position showed limited spatial dispersion within the observed 3SD range, the relationship between PP occurrence and limb dominance should be interpreted cautiously. In most participants, the predominant PP occurrence was observed on the dominant limb (63–97% of trials); however, this finding represents a preliminary observation that requires confirmation in larger cohorts.

The manual determination of coordinates from color maps may represent a practical approach when numerical export is unavailable. Low SEM values and the absence of systematic bias on the Bland–Altman diagrams (Figure 3 suggest that this procedure provides consistent coordinate estimation under the applied methodological conditions, provided that a uniform methodology (40 × 40 cm reference frame) is maintained.

The main limitation of this study is the small sample size (n = 5), which restricts the generalizability of the findings and does not allow conclusions regarding population-level reliability or clinical applicability of the device. Although 30 repeated measurements per participant enabled detailed assessment of intra-individual variability, this approach cannot compensate for the limited number of participants. In addition, the small cohort did not allow meaningful subgroup analyses according to sex, age, or other demographic and anthropometric characteristics. Although participant characteristics such as BMI, foot size, lower-limb dominance, habitual physical activity, and baseline foot characteristics were documented, the limited sample size did not permit evaluation of their individual influence on plantar pressure parameters, center of pressure behavior, or measurement repeatability. Previous studies have shown that these diversity-related factors may influence plantar pressure distribution and postural behavior and should therefore be considered when interpreting pedobarometric data [8]. Consequently, the presented results should be interpreted as preliminary evidence of intra-session repeatability under controlled laboratory conditions. Future studies involving larger and more diverse populations are required to investigate the influence of demographic and anthropometric characteristics on plantar pressure parameters, center of pressure behavior, peak pressure localization, measurement repeatability, and their potential clinical relevance. 

## 5. Conclusions

The results indicate that the Sidas Press-Cam pedobarometric platform, in conjunction with the proposed methodological procedure, provides stable and reproducible values for the contact area of both feet, as well as for the right and left limbs individually. Although values for peak and mean pressure exhibit fluctuations, the device is suitable for both clinical and research purposes. To achieve maximum precision in the assessment of pressure peaks, we recommend analyzing the averages of multiple repeated measurements. These findings are consistent with the available literature and support the use of multiple measurements as a standard approach in static pedobarometry.

The measurements demonstrate that the device provides repeatable and consistent center of pressure (CoP) data, capturing the expected medio-lateral variations and the positional stability of the peak pressure (PP) in the subjects. The findings indicate that, under controlled laboratory conditions, repeated measurements using the Sidas Press-Cam platform showed consistent intra-session patterns for plantar pressure parameters, CoP localization, and PP position. However, due to the limited sample size, these results should be considered preliminary and require confirmation in larger populations before conclusions regarding clinical applicability can be drawn.

## Figures and Tables

**Table 1:** Descriptive statistics of plantar loading parameters (mean ± SD and median).

Subject	Subject 1	Subject 2	Subject 3	Subject 4	Subject 5
PP (gcm^−2^)					
mean	806.20	933.83	835.47	797.70	756.23
SD	64.23	72.12	48.27	56.50	66.43
median	819.50	929.00	842.50	783.00	753.50
MP (gcm^−2^)					
mean	332.53	355.80	324.33	327.50	301.40
SD	11.06	11.80	21.63	13.32	13.38
median	332.00	359.00	324.00	326.50	301.00
AC (cm^2^)					
mean	210.73	239.20	247.73	266.13	186.13
SD	6.91	8.13	16.61	10.81	8.40
median	211.00	237.00	247.00	266.50	186.00
ACl (cm^2^)					
mean	104.97	118.60	125.43	130.47	94.03
SD	3.80	4.39	7.74	6.61	4.45
median	105.00	118.00	124.00	131.00	93.50
ACr (cm^2^)					
mean	105.77	120.60	122.30	135.67	92.10
SD	5.39	5.36	10.06	5.27	4.76
median	105.50	120.50	121.00	136.00	92.00

**Table 2:** Coordinates of the center of pressure (CoP) in cm (mean ± SD).

Subject	CoPt (cm)		CoPr (cm)		CoPl (cm)	
	x	y	x	y	x	y
Subject 1	9.05 ± 0.68	10.57 ± 0.62	17.47 ± 1.14	10.25 ± 0.74	0.59 ± 0.62	10.87 ± 0.57
Subject 2	11.08 ± 0.91	11.91 ± 0.44	20.09 ± 0.95	12.14 ± 0.52	2.62 ± 0.56	11.69 ± 0.63
Subject 3	11.08 ± 0.68	10.64 ± 0.52	19.43 ± 0.77	10.84 ± 0.64	3.30 ± 0.40	10.47 ± 0.53
Subject 4	10.20 ± 0.71	12.40 ± 0.53	18.88 ± 1.33	13.04 ± 0.58	1.12 ± 0.52	11.69 ± 0.64
Subject 5	9.65 ± 0.67	10.84 ± 0.45	17.06 ± 0.98	10.67 ± 0.55	2.60 ± 0.56	11.01 ± 0.48

**Table 3:** Repeatability of static pedobarometric measurements expressed by ICC, standard error of measurement (SEM), and limits of agreement (LoA).

Parameter	ICC(2, 1)	ICC(2, k)	SEM	95% LoA (Mean Difference ± 1.96 SD)
PP (gcm^−2^)	0.53	0.97	58.69	−186.6 to +150.0
MP (gcm^−2^)	0.63	0.98	13.82	−37.3 to +43.5
AC (cm^2^)	0.90	0.996	9.58	−31.7 to +27.2
ACl (cm^2^)	0.88	0.995	5.04	−16.4 to +14.4
ACr (cm^2^)	0.87	0.995	5.89	−19.1 to +16.5

## Data Availability

Dataset available on request from the authors.

## References

[1] Alfaro-Santafé J.-V., Gómez-Bernal A., Almenar-Arasanz A.-J., Alfaro-Santafé J. (2021). Reliability and repeatability of the footwork plantar pressure plate system. J. Am. Podiatr. Med. Assoc..

[2] Izquierdo-Renau M., Pérez-Soriano P., Ribas-García V., Queralt A. (2017). Intra and intersession repeatability and reliability of the S-Plate^®^ pressure platform. Gait Posture.

[3] Fullin A., Caravaggi P., Picerno P., Mosca M., Caravelli S., De Luca A., Lucariello A., De Blasiis P. (2022). Variability of postural stability and plantar pressure parameters in healthy subjects evaluated by a novel pressure plate. Int. J. Environ. Res. Public Health.

[4] De Blasiis P., Caravaggi P., Fullin A., Leardini A., Lucariello A., Perna A., Guerra G., De Luca A. (2023). Postural stability and plantar pressure parameters in healthy subjects: Variability, correlation analysis and differences under open and closed eye conditions. Front. Bioeng. Biotechnol..

[5] Cobos-Moreno P., Astasio-Picado Á., Martínez-Nova A., Sánchez-Rodríguez R., Escamilla-Martínez E., Gómez-Martín B. (2022). The Podoprint^®^ plantar pressure platform: Evaluation of reliability and repeatability, and determination of the normality parameters. J. Tissue Viability.

[6] Lalande X., Vie B., Weber J.P., Jammes Y. (2016). Normal values of pressures and foot areas measured in the static condition. J. Am. Podiatr. Med. Assoc..

[7] Mesci E. (2023). Pedobarographic evaluations in physical medicine and rehabilitation practice. Turk. J. Phys. Med. Rehabil..

[8] Mirando M., Pavese C., Pingue V., Sozzi S., Nardone A. (2025). Can plantar pressure distribution during gait be estimated from quiet stance in healthy individuals?. J. Funct. Morphol. Kinesiol..

[9] Vyawahare M., Govilkar S. (2022). Fake profile recognition using profanity and gender identification on online social networks. Soc. Netw. Anal. Min..

[10] Thakur N., Cui S., Khanna K., Knieling V., Duggal Y.N., Shao M. (2023). Investigation of the Gender-Specific Discourse about Online Learning during COVID-19 on Twitter Using Sentiment Analysis, Subjectivity Analysis, and Toxicity Analysis. Computers.

[11] Rodríguez-Rubio P.R., Bagur-Calafat C., López-de-Celis C., Bueno-Gracía E., Cabanas-Valdés R., Herrera-Pedroviejo E., Girabent-Farrés M. (2020). Validity and reliability of the Satel 40 Hz stabilometric force platform for measuring quiet stance and dynamic standing balance in healthy subjects. Int. J. Environ. Res. Public Health.

[12] Xu C., Wen X.-X., Huang L.Y., Shang L., Cheng X.-X., Yan Y.-B., Lei W. (2017). Normal foot loading parameters and repeatability of the Footscan^®^ platform system. J. Foot Ankle Res..

[13] Bruyneel A.-V., Mesure S., Reinmann A., Sordet C., Venturelli P., Feldmann I., Guyen E. (2022). Validity and reliability of center of pressure measures to quantify trunk control ability in individuals after stroke in subacute phase during unstable sitting test. Heliyon.

[14] Giacomozzi C. (2010). Appropriateness of plantar pressure measurement devices: A comparative technical assessment. Gait Posture.

[15] Putti A.B., Arnold G.P., Cochrane L., Abboud R.J. (2007). The Pedar in-shoe system: Repeatability and normal pressure values. Gait Posture.

[16] Quijoux F., Nicolaï A., Chairi I., Bargiotas I., Ricard D., Yelnik A., Oudre L., Bertin-Hugault F., Vidal P.P., Vayatis N. (2021). A review of center of pressure (COP) variables to quantify standing balance in elderly people: Algorithms and open-access code. Physiol. Rep..

